# Records of threatened bird and mammal species in Mato Grosso do Sul State, Brazil

**DOI:** 10.1016/j.dib.2018.02.052

**Published:** 2018-02-23

**Authors:** Sylvia Torrecilha, Jose Manuel Ochoa-Quintero, Rudi Ricardo Laps, Danilo Bandini Ribeiro

**Affiliations:** a Programa de Pós Graduação em Ecologia e Conservação, Universidade Federal de Mato Grosso do Sul, CP 79070-900, P.O. Box 549, Campo Grande, MS, Brazil; b Instituto de Investigación de Recursos Biológicos Alexander von Humboldt, Bogotá, colombia; c Instituto de Biociências, Universidade Federal de Mato Grosso do Sul, CP 79070-900, P.O. Box 549, Campo Grande, MS, Brazil

**Keywords:** Threatened species, Protected areas, Database, Brazil

## Abstract

We conducted a detailed review of threatened bird and mammal occurrence records obtained from surveys across Mato Grosso do Sul, midwestern region of Brazil which has an extent of 357,145 km^2^, aiming to support environmental and biodiversity conservation initiatives, as strategic plans to protect threatened species in this region. We included all records of species categorized as threatened by the Brazilian and global red list of threatened species. We collected 760 records of threatened birds and mammals in Mato Grosso do Sul State, with 319 records of 40 bird’s species and 441 records of 24 mammal’s species. The status of the 40 bird species under de Brazilian threat category were as follow: 1 Critically Threatened (CR), 6 Endangered (EN), 11 Vulnerable (VU), 11 Near Threatened (NT), and 11 species only in the IUCN red list. Under the IUCN category for the bird´s species, were as follow: 3 EN, 13 VU, 18 NT, 5 Least Concern (LC) and 1 taxon has not yet been assessed for the IUCN Red List. Regarding mammal’s species under the Brazilian threat category were as follow: 2 EN, 18 VU, 2 NT and 1 only in the IUCN red list. Under the IUCN status the species ranged from 2 EN, 6 VU, 10 NT, and 6 LC. Each record identified corresponds to the existence of at least one occurrence of threatened birds or mammals in a particular region. The records of threatened species belongs to the three biomes in the state: 269 mammal’s records and 147 bird’s records from Cerrado (Neotropical Savanna) biome, 117 mammal’s records and 162 bird’s records from Pantanal (Wetland) biome, and 55 mammal’s records and 10 bird’s records from Atlantic Forest biome. In addition, we also included in the dataset environmental information where each record was obtained. Supplementary Files 1- Records of Threatened Mammals_MS_Brazil and Supplementary File 2. Records of Threatened Birds of_MS_Brazil

**Specifications table**

**Table:** 

Subject area	Environmental Science
More specific subject area	Nature and Landscape Conservation
Type of data	Table
How data was acquired	We conducted a detailed review of threatened bird and mammal occurrence records obtained from surveys across Mato Grosso do Sul, midwest of Brazil. We included all records of species categorized as threatened by the Brazilian and global red list (IUCN) of threatened species. The Brazilian list of threatened species were obtained from MMA regulations nº 444/2014 and nº 43/2014 (Ministério do Meio Ambiente, Instituto Chico Mendes de Conservação da Biodiversidade, 2014), via the website (http://www.icmbio.gov.br/portal/faunabrasileira/lista-de-especies) and for those species classified as Near Threatened (NT) via the website (http://www.icmbio.gov.br/portal/faunabrasileira/lista-de-especies-dados-insuficientes) and IUCN Red List of Threatened Species, from (http://www.iucnredlist.org/).
Data format	Tables that contains summarized georeferenced records of threatened birds and mammals’ species in MS State, southwestern Brazil. We included all records of species categorized as threatened by the Brazilian [2], [3] and global red list [4] of threatened species. These tables still contains specific relevant species information, including number of records, common name, at Brazil and IUCN threatened category, locality, local environmental characteristics and vegetation type, biome, river basin, country and state, county and reference.
Experimental factors	To build GIS database model we generated tables that contains the geographic coordinates standardized in decimal degrees, which were processed in ArcGIS software. With this information, we have created a map including all the occurrence records for each species of threatened mammals and birds at the state level. The cartographic maps data sources used to verifying the occurrence were hydrography and roads. All records with possible problems of field reliability were discarded: secondary source of information, no field sampling effort, inconsistencies with the known distribution of the species, and also events with inaccurate geographic locations.
Experimental features	
Data source location	Mato Grosso do Sul, midwestern region of Brazil which has an extent of 357,145 km^2^. The coordinates systems in the tables are standardized in decimal degrees and the reference datum is SAD 69. Coordinates: -54,23 and -24,23 and -53,64 and -17,31 Latitude; -58,11 and -19,75 and -50,91 and -19,57 Longitude. Dates of source publications range from 2000 to 2015.
Data accessibility	Supplementary Files 1- Records of Threatened Mammals _MS_Brazil and Supplementary File 2. Records of Threatened Birds of_MS_Brazil.

**Value of the data**

• Disseminate reliable data and information about threatened species occurrence in this case Brazil, south west with especial focus on its biogeographical distributions.
• Provide biodiversity data in decision-making models focusing on tropical conservation strategies.
• Include many cinegetic and key species, that may be effective surrogates for occurrence of a much broader set of species to signal priority areas for conservation
• Make available precise information to be incorporated directly in the public decision-making policies, understanding and addressing the gaps that prevent a direct application

## 1 Data

This article presents a detailed review of threatened bird and mammal occurrence records obtained from surveys across Mato Grosso do Sul, midwestern of Brazil, aiming to support environmental and biodiversity conservation initiatives, as strategic plans to protect threatened species in this region. We included all records of species categorized as threatened by the Brazilian [2], [3] and global red list [4]. The Brazilian list of threatened species were obtained from MMA regulations Nº 444/2014 and Nº 43/2014 [1], via the website (http://www.icmbio.gov.br/portal/faunabrasileira/lista-de-especies) and for those species classified as Near Threatened (NT) via the website (http://www.icmbio.gov.br/portal/faunabrasileira/lista-de-especies-dados-insuficientes) and IUCN Red List of Threatened Species from (http://www.iucnredlist.org/).

We collected 760 records of threatened birds and mammals in Mato Grosso do Sul State, with 319 records of 40 bird’s species and 441 records of 24 mammal’s species. The status of the 40 bird species under de Brazilian threat category were as follow: 1 specie Critically Threatened (CR), 6 species Endangered (EN), 11 species Vulnerable (VU), 11 species Near Threatened (NT), and 11 species only in the IUCN red list. Under the IUCN category for the bird´s species, were as follow: 3 species EN, 13 species VU, 18 species NT, 5 species Least Concern (LC) and 1 taxon has not yet been assessed for the IUCN Red List. Regarding mammal’s species under the Brazilian threat category were as follow: 2 species EN, 19 species VU, 2 species NT and 1 only in IUCN red list. Under the IUCN status the species ranged from 2 EN, 6 VU, 10 NT, and 6 LC. Each record identified corresponds to the existence of at least one occurrence of threatened birds or mammals in a particular region.

The records of threatened species belongs to the three biomes in the state: 269 mammal’s records and 147 bird’s records from Cerrado (Neotropical Savanna) biome, 117 mammal’s records and 162 bird’s records from Pantanal (Wetland) biome, and 55 mammal’s records and 10 bird’s records from Atlantic Forest biome. In addition, we also included in the dataset environmental information where each record was obtained. Supplementary Files 1- Records of Threatened Mammals_MS_Brazil and Supplementary File 2. Records of Threatened Birds_MS_Brazil.

## 2 Experimental design, materials and methods

### 2.1 Study area

This study focused on Mato Grosso do Sul state, in the midwestern region of Brazil, which has an extent of 357,145 km^2^. The state extends from the international borders with Paraguay and Bolivia, to borders with Mato Grosso, Goiás, São Paulo and Paraná states in Brazil. The state is limited by the Paraguay River to the south, southwest and north and Parana River to the east. It consists of two continuous geographic areas, formed by the Pantanal plain, a large wetland with much natural vegetation, (~89,000 km^2^), and a plateau, covered originally by neotropical savana, and dry forest.

### 2.2 Data collection

We conducted a detailed literature review of threatened bird and mammal occurrence records obtained from surveys across Mato Grosso do Sul state. We choose these two taxonomic groups, because (i) they are widely used as environmental indicators, (ii) data are relatively abundant about their occurrence (iii) they include many species listed as threatened and (iv) they include many cinegetic and key species, that may be effective surrogates for occurrence of a much broader set of species to signal priority areas for conservation [6]. The literature review (database) included records from sources including Machado et al. [5], protected areas management plans, environmental impact studies (EIA, IMASUL / MS) and self-monitoring plans from Mato Grosso do Sul Environment Institute over 2008–2015. To compile published articles on fauna of the state, we used the search system of the ISI Web of Knowledge data database with the following keywords: Mato Grosso do Sul AND OR mammals * OR mammal OR *bird OR birds.

For classification of birds we used the Brazilian Ornithological Records Committee list [7], for mammals the overall taxonomic nomenclature follows Paglia et al. [8], which incorporates Wilson and Reeder [9]. All records with possible problems of reliability were discarded (secondary source of information, no field sampling effort, inconsistencies with the known distribution of the species) and events with inaccurate geographic locations.

**Fig. 1:**
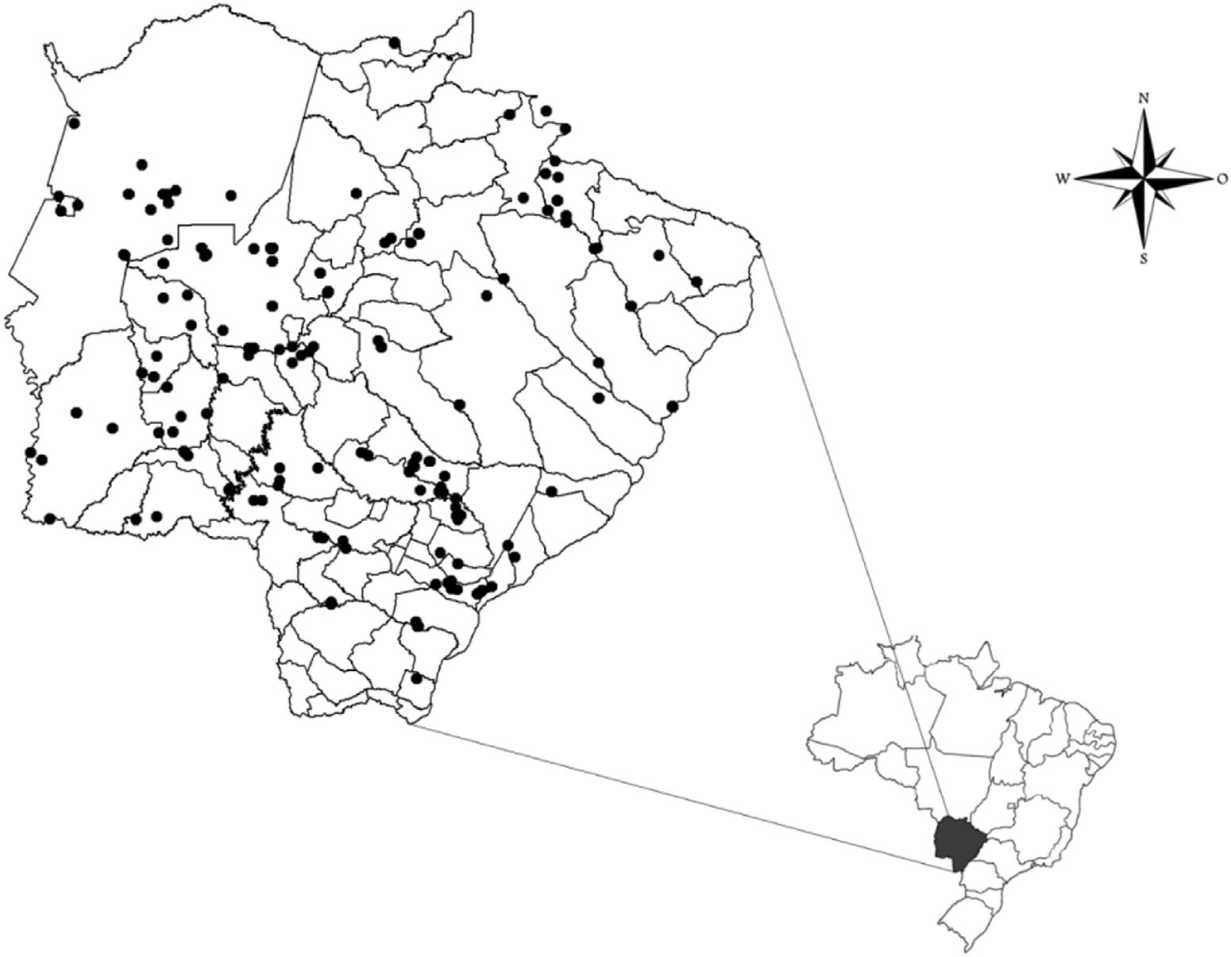
Map with known occurrences of species of mammals and birds in Mato Grosso do Sul State, Brazil.

To build the GIS database model we generated tables that contains the geographic coordinates standardized in decimal degrees, datum SAD 69, which were processed in ArcGIS software (version 10.0). With this information, we have created a map including all the occurrence records of each species of threatened birds and mammals at the state level (Fig. 1).

## References

[1] MMA, Environmental Ministry, ICMBio. Brazilian Red List of Threatened Species. 〈http://www.icmbio.gov.br/portal/faunabrasileira/lista-de-especies〉 (accessed 22 September 2015), 2015.

[2] MMA, Environmental Ministry. Portaria MMA Nº 444. 〈http://www.icmbio.gov.br/portal/faunabrasileira/2741-lista-de-especies-ameacadas-saiba-mais.html〉, 2014 (accessed 22 September 2015).

[3] MMA, Environmental Ministry. Portaria MMA Nº 43. 〈http://www.icmbio.gov.br/portal/faunabrasileira/lista-de-especies-dados-insuficientes〉, 2014 (accessed 22 September 2015).

[4] IUCN, International Union for Conservation of Nature, IUCN Red List of Threatened Species. 〈http://www.iucnredlist.org/technical-documents/spatial-data〉 (accessed 22 September 2015), 2015.

[5] Machado A.B., Drummond G.M., Paglia A.P. (2008). Livro vermelho da fauna brasileira ameaçada de extinção.

[6] Gaston K.J. (1991). Regional numbers of insect and plant species. Funct. Ecol..

[7] Piacentini V.Q., Aleixo A., Agne C.E., Maurício G.N., Pacheco J.F., Bravo G.A., Brito G.R.R., Naka L.N., Olmos F., Posso S., Silveira L.F., Betini G.S., Carrano E., Franz I., Lees A.C., Lima L.M., Pioli D., Schunck F., Amaral F.R., Glayson A.B., Cohn-Haft M., Figueiredo L.F.A., Straube F.C., Cesari E. (2015). Annotated checklist of the birds of Brazil by the Brazilian Ornithological Records Committee. Rev. Bras. Ornitol..

[8] A.P. Paglia, G.A.B. Fonseca, A.B. Rylands, G. Herrmann, L.M.S. Aguiar, A.G. Chiarello, Y.L.R. Leite, L.P. Costa, S. Siciliano, M.C.M. Kierulff, S.L. Mendes, V.C. Tavares, R.A. Mittermeier, J.L. Patton, Lista Anotada dos Mamíferos do Brasil/ Annotated Checklist of Brazilian Mammals. 2ed. Occasional Papers in Conservation Biology 6 (2012)1–76, 2012.

[9] Wilson D.E., Reeder D.M. (2005). Mammal Species of the World – a Taxonomic and Geographic Reference.

