# Supplementary material for: Records of threatened bird and mammal species in Mato Grosso do Sul State, Brazil
Source: Data Brief. 2018 Feb 23;17:1326–30. doi: 10.1016/j.dib.2018.02.052 (PMC5988451; doi:10.1016/j.dib.2018.02.052)
Supplement: Supplementary file 1 — Supplementary material [file mmc1.docx]

Dear Editor

We declare that there is no potential conflict of interest in the support and elaboration of this manuscript: **Records of Threatened Bird and Mammal Species in Mato Grosso do Sul State, Brazil”.** This research is part of a thesis content of the postgraduate program, and all investigators worked closely and integrated ; in the collection, analysis, interpretation of data, in the writing of the report; and finally in the decision to submit the paper for publication. We also declare that there is no further conflict between the Institution in charge of this research, Federal University of Mato Grosso do Sul, Ecology and Conservation Postgraduate Program (PPGEC) and those institutions that provided support to collect and systematize species records, Instituto de Meio Ambiente do Mato Grosso do Sul and Biota-MS.

Sylvia Torrecilha

Corresponding author
